# Complicated Odontogenic Infections at 2 District Hospitals in Tonkolili District, Sierra Leone: Protocol for a Prospective Observational Cohort Study (DELAY)

**DOI:** 10.2196/33677

**Published:** 2021-12-13

**Authors:** Hanna M J L Hazenberg, Jan Henk Dubbink, Issa Sesay, Tom Versteege, Hassan Bangura, Louise K Hoevenaars, Abdul M Falama, Heleen M Koudijs, Rosa Roemers, Emmanuel B Bache, Emil F Kelling, Frieder Schaumburg, Fred K L Spijkervet, Martin P Grobusch

**Affiliations:** 1 Center for Tropical Medicine and Travel Medicine Department of Infectious Diseases, Division of Internal Medicine Amsterdam University Medical Center, Location Amsterdam Medical Center Amsterdam The Netherlands; 2 Masanga Medical Research Unit Masanga Hospital Masanga Sierra Leone; 3 KIT Royal Tropical Institute Amsterdam The Netherlands; 4 Lion Heart Medical Centre Yele Sierra Leone; 5 District Health Medical Team District Medical Office Magburaka Sierra Leone; 6 Institute of Medical Microbiology University Hospital Münster Münster Germany; 7 Department of Oral and Maxillofacial Surgery University Medical Center Groningen Groningen The Netherlands

**Keywords:** complicated odontogenic infection, dental abscess, deep neck space infection, Ludwig angina, necrotizing fasciitis, prospective observational cohort study

## Abstract

**Background:**

Deficits in global oral health care are paramount, and complications of odontogenic infections constitute a considerable global health problem, particularly in low-income countries. A high mortality rate has been observed for patients who have been admitted with complicated odontogenic infections to our facilities in Tonkolili District, Sierra Leone, although exact data have not been published yet. Data regarding who in this region is at risk and why are lacking.

**Objective:**

The Dental Abscess Study (DELAY) aims to prospectively investigate morbidity and mortality from complicated dental abscesses and to analyze patients’ characteristics and microbial findings to examine predisposing factors for poor outcomes. In particular, the incidence and the clinical and microbial characteristics of complicated odontogenic infections, as well as the sociodemographic data and comorbidities of affected patients, will be studied to develop improved management algorithms based on circumstance-specific factors.

**Methods:**

Patients who present with complicated dental infections requiring hospital admission in Masanga Hospital or Lion Heart Medical Centre will be consecutively selected for possible inclusion in the study (starting on September 4, 2021) over a study period of 1 year, and individual routine follow-ups will be conducted at least 3 months after discharge. The results of standardized questionnaires will be obtained, and clinical measurements as well as medical photos will be taken. Standard laboratory tests (eg, full blood count and HIV status tests) will be performed, and pus specimens will be examined. Local treatment guidelines will be adhered to, and data on medical and surgical treatment as well as data on outcomes will be collected. The study results will be reported according to the STROBE (Strengthening the Reporting of Observational Studies in Epidemiology) criteria. Routine follow-ups will take place at 1 and 3 months postdischarge.

**Results:**

The DELAY protocol was endorsed by the Masanga Medical Research Unit’s Scientific Review Committee on June 16, 2021, and ethical approval was granted on July 5, 2021, by the Sierra Leone National Ethics Committee. The funding of the budgeted study costs was approved by Dental Health International Netherlands in August 2021. The projected start date of data collection was September 4, 2021, and the study period will most likely last for 1 year. As such, data collection is expected to be complete in November 2022.

**Conclusions:**

The aim of our prospective observational cohort study is to gain more knowledge about complicated odontogenic infections in Tonkolili District, Sierra Leone, to further improve treatment strategies.

**International Registered Report Identifier (IRRID):**

DERR1-10.2196/33677

## Introduction

### Background

Globally, one of the main reasons for visiting a health care facility is dental infection [1,2]. Odontogenic infections can be the result of dental caries, periodontitis, pericoronitis, or complications from dental procedures and can result in the formation of dental abscesses [3]. Both children and adults can be affected by odontogenic infections, and underprivileged groups are especially at risk [4]. Appropriate dental care services are lacking in most low-income countries, and shortages of human resources as well as unmet dental health needs have been reported from Sierra Leone [5,6]. If odontogenic infections are left untreated, dental abscesses can form, which can result in complications such as Ludwig angina, retropharyngeal spread, mediastinitis, necrotizing fasciitis, osteomyelitis, and intracranial abscesses. These complications are associated with high mortality rates [7-10]. The first therapy of choice for abscesses resulting from deep neck space infections (DNSIs) is incision and drainage treatment, and antibiotics are a cornerstone of DNSI treatment [11]. However, due to the inappropriate, widespread use of broad-spectrum antibiotics, pathogens that cause oral infections have become more resistant to antibiotic treatment [12]. A flowchart specifying the criteria for admitting and identifying patients with infections that have a high risk of secondary complications was proposed by one study [13]. Another study developed a risk assessment scale and management algorithm for DNSIs [14]. Although the management algorithm was deemed useful for that particular cohort, which was recruited from a high-income country, it does not seem to be feasible for low- and middle-income country environments where advanced medicotechnological instruments, such as fiberscopes, are not readily available. To the best of our knowledge, up-to-date data on the prevalence, characteristics, and management of complicated dental abscesses in Sierra Leone have not been published yet. As such, our study—the Dental Abscess Study (DELAY)—will prospectively investigate morbidity and mortality from complicated dental abscesses and analyze patients’ characteristics and microbial findings to identify predisposing factors for major complications.

### Hypothesis and Research Objectives

In our study, we hypothesized that extensive odontogenic infections are a frequent and potentially life-threatening clinical problem in Tonkolili Province, Sierra Leone. Current diagnosis, treatment, and outcome management methods require improvement. As such, our study will address the general and specific objectives provided in Textboxes 1 and 2.

**General objectives.**

**Objectives**

- To study the incidence of complicated odontogenic infections at 2 district hospitals in rural Sierra Leone
- To study the clinical and microbiological characteristics of severe odontogenic infections
- To study the comorbidities, complications, and outcomes of extensive odontogenic infections
- To develop improved management algorithms based on circumstance-specific factors

**Specific objectives.**

**Objectives**

- To assess the prevalence and severity of cases presenting with complicated odontogenic infections requiring hospital admission
- To describe the study population based on sociodemographic information and clinical presentation
- To determine the causative agents and the resistance spectrum of complicated odontogenic infections
- To define risk factors of the severity and sequelae of odontogenic infections (ie, dental abscess formation, cellulitis, phlegmon, necrotizing fasciitis, osteomyelitis, mediastinitis, and the need for cricothyroidotomy) based on sociodemographic information, clinical presentation, and HIV status
- To correlate the physical examination findings with disease severity and outcomes
- To correlate the microbial findings with disease severity and outcomes
- To retrospectively assess antimicrobial therapy appropriateness and to develop locally relevant antibiotic treatment guidelines for odontogenic infections
- To propose improved health service algorithms that match locally available resources to patients’ needs (ie, after the primary data analysis)

## Methods

### Study Design

The DELAY is a prospective longitudinal cohort study that will be conducted at 2 rural district hospitals in Sierra Leone and will include any cases that are eligible for inclusion, as per the inclusion and exclusion criteria. The study will have a 1-year enrollment period between September 2021 and August 2022. Study participants will routinely be followed up for 3 months.

### Disclaimer

It is important to indicate that standard local treatment guidelines will be adhered to, regardless of whether patients decide to participate in the DELAY. Given that there will be no interference with the routine treatment modalities, oral consent was considered to be appropriate. That notwithstanding, written informed consent (or thumb-printed consent from illiterate participants) will be documented in a separate form. This consent encompasses consent for the initial examination and all follow-up examinations, including the taking of medical photos. At any point during the study, consent can be withdrawn.

### Study Sites

Masanga Hospital is a 120-bed general district hospital that serves a population of around 440,000 people. Masanga is a remote town located in the Kholifa Rowala Chiefdom of the Tonkolili District in Sierra Leone’s Northern Province. The hospital offers inpatient and outpatient services related to internal medicine, pediatrics, surgery, and obstetrics and gynecology and is transitioning from a nongovernmental organization–supported hospital to a fully governmental hospital.

Lion Heart Medical Centre (LHMC) is a 70-bed general district hospital in Yele—a town in the remote south of the Tonkolili District. LHMC serves a catchment population of around 100,000 people who are mainly from the Gbonkolenken, Valunia, and Kamajei chiefdoms. LHMC offers inpatient and outpatient services related to internal medicine, pediatrics, surgery, and obstetrics and gynecology. LHMC too is a nongovernmental organization–supported hospital that is transitioning to become a government-supported hospital in the coming years.

### Population

The study population will be selected consecutively from patients who present with complicated odontogenic infection requiring in-hospital admission to 1 of the 2 district hospitals in Tonkolili District—Masanga Hospital or LHMC—from September 4, 2021, to August 31, 2022 (this period is subject to prolongation depending on the number of study participants). Consequently, the study population will most likely consist of, but will not be limited to, Sierra Leone citizens from the Tonkolili District.

### Inclusion Criteria

In order to be eligible to participate in this study, a patient must meet the following inclusion criteria: (1) the in-hospital admission of any patient due to the clinical suspicion of a complicated odontogenic infection (eg, DNSI, necrotizing fasciitis, cellulitis, dental abscess formation, osteomyelitis, and mediastinitis) and (2) the provision of documented informed consent by a patient or a legal representative.

### Exclusion Criteria

A potential subject who meets any of the following criteria will be excluded from participating in this study: (1) a swollen neck resulting from any cause other than odontogenic infection (eg, goiters, congenital cysts, and trauma), (2) oral and neck infections resulting from any cause other than odontogenic infection (eg, isolated peritonsillar cellulitis, tonsillitis, isolated osteomyelitis, trauma, or infection after trauma), and (3) the inability of a patient or a legal representative to provide informed consent.

### Sample Size

A formal case size calculation has not been conducted, as there is no effect-modifying intervention that can be compared against a therapeutic (clinical) gold standard. The DELAY is an observational study for informing future larger studies on odontogenic infection. We aim to include at least 300 patients from both locations combined. However, we propose the inclusion of 350 patients in the study to account for possible missing data and losses to follow-up.

### Data Collection

On admission, study information will be provided to individuals (or their primary caretakers) who are apparently eligible for inclusion, and informed consent will be sought. Subjects who consent and are found to be eligible after checking the inclusion and exclusion criteria will be assigned unique participant identification numbers (PINs). In Masanga Hospital (in the village of Masanga), the first patient will be assigned the PIN “M001,” the second patient will be assigned the PIN “M002,” and so forth. In LHMC (in the town of Yele), the first patient will be assigned the PIN “Y001,” the second patient will be assigned the PIN “Y002,” and so forth.

After PIN assignment, a standardized questionnaire will be completed. The questionnaire contains questions about patients’ characteristics (eg, age, sex, past medical history, and socioeconomic status), clinical symptoms (eg, the start and duration of complaints), and health behaviors (eg, consultation with a traditional healer and dental care). A physical extraoral and intraoral examination will be performed at and during admission and at discharge. These will include measurements of the interlobar distance [15], Adam distance [16], and interincisal distance at maximal mouth opening [16,17] (Table 1).

Details on the clinical conditions of patients upon admission (eg, vital signs, airway patency, and AVPU [Alert, Responsive to Verbal Stimulus, Responsive to Pain Stimulus, Unresponsive] scale scores), as well as suspected complications (eg, sepsis, cervical necrotizing fasciitis, and mediastinitis), will be noted. If a patient has provided consent, a medical photo will be taken. Standard laboratory testing will be conducted depending on the availability at the study site and will include hemoglobin tests (Masanga and Yele), white blood cell count tests (Masanga and Yele), malaria rapid testing, hepatitis B surface antigen tests, and HIV testing (Masanga and Yele).

If applicable, a pus swab will be taken and sent to the laboratory at the University Hospital of Münster, Germany, for culture and bacterial cell count tests as well as antibiotic susceptibility testing. Moreover, a pure pus sample will be taken and stored locally in a freezer. A bacteriology lab is under construction at the Masanga site—a development that has been delayed due to COVID-19 epidemic circumstances. Provided that the on-site bacteriology laboratory becomes operational during the study period, which is likely to be the case, the samples will be tested there, and the results will be validated at the Münster collaboration site. If blood culture material becomes available at the local level during the study period, blood cultures will be collected. Treatment will be provided in accordance with standard local treatment guidelines; we will not wait for antibiotic susceptibility results before the start of antibiotic therapy, given the long shipping times. As the local laboratory will most likely not be certified during the study periods, and as the results from the Münster reference laboratory will only become available after an individual patient’s acute treatment period, laboratory results will not be used to modify treatment.

Data on treatment will be collected on the date of admission (eg, the use of antibiotics and planned surgeries) and on the day(s) of surgery (ie, if a surgery will be performed). Additionally, several outcome parameters will be measured on the day of admission; on postoperative days 1, 2, and 7 (in cases where surgery has been performed); on discharge; and at both routine follow-up visits. These will include the interlobar distance, the maximal interincisal mouth opening capacity, and the Adam distance. On discharge, additional data on outcomes will be collected (eg, the state of wound closure, the presence of facialis paresis, and mortality), and medical photos will be taken.

Follow-up visits will take place on postdischarge weeks 4 and 12. If necessary, additional follow-up visits will be conducted. A standardized questionnaire regarding current symptoms will be taken at follow-up visits, during which a physical examination will be performed and a photo will be taken.

Further specifics on which parameters will be recorded during admission, discharge, and follow-up can be found on the case record form in this study protocol (Multimedia Appendix 1).

**Table 1:** Clinical measurement definitions.

Measurement	Anatomic site of measurement	Time of measurement	Tools used
Interlobar distance [15]	From the tip of the tragus to the gonion and to the contralateral tip of the tragus	Upon admission Postoperative days 1, 2, and 7 Follow-up weeks 4 and 12	Tape measure
Maximal interincisal mouth opening capacity or trismus [16,17]	From the incisal edge of the maxillary central incisor to the incisal edge of the mandibular central incisor	Upon admission Postoperative days 1, 2, and 7 Follow-up weeks 4 and 12	Vernier caliper
Adam distance [16]	From the laryngeal cartilage to the vermilion border of the lower lip at rest	Upon admission Postoperative days 1, 2, and 7 Follow-up weeks 4 and 12	Tape measure

### Data Capture and Entry

Paper versions of the informed consent forms will be filled out and stored on-site upon admission. Data will be collected and stored on-site on paper case record forms by trained research staff. During the data collection phase of the study, all variables will be entered on a research computer by using Microsoft Access at each separate study site (offline database). Data will be double entered by 2 different researchers on 2 separate forms and subsequently matched to identify errors.

All medical photos taken during the study will be encoded by using the following file name format: “PIN_contact moment.” In the *contact moment* field, 1 of 4 labels will be used, as follows: “A” (admission), “D” (discharge), “F1” (follow-up visit 1 after 1 month), and “F2” (follow-up visit 2 after 3 months). An example file name of a photo that is taken upon the admission of a theoretical study participant in the Masanga study site would be “M005_A.png.” Medical photos will be stored on a hard drive during the data collection period. After the study period, all study data will be stored for a minimum of 15 years in 1 database at the Masanga Medical Research Unit (MMRU).

### Data Analysis

Microsoft Excel and SPSS (IBM Corporation) will be used for the analysis of the data. An exploratory analysis of the variables and descriptive statistics of the data will be carried out. Study parameters will be assessed from an etiologic perspective. For data consistency, certain groups might be excluded from the data analysis (eg, neonates). The description of the distribution of each variable will be made based on measures of central tendency (mode, mean, and median) and measures of dispersion (variance, SD, and maximum and minimum values). Pearson chi-square tests and Kaplan-Meier curves will be used to analyze the study objectives. Logistic regression analyses as well as Cox regression models will be conducted to assess the predictive value of patients’ characteristics and microbial findings on outcomes.

### Ethical Considerations

Patients will be approached by the researchers after admission to the hospital and will be asked to provide informed consent after they have received and studied the patient information (Multimedia Appendix 2). This consent will include permission to publish anonymized (nonidentifiable) picture material. Since treatment will be performed according to the local guidelines, participation in the study will not influence the treatment that patients receive. Informed consent files (Multimedia Appendix 3) will be kept at the MMRU and at LHMC, as will the patient-reported questionnaires. Study participants can leave the study at any time for any reason if they wish to do so, without any negative consequences for the care provided. The investigator can decide to withdraw a participant from the study for urgent medical reasons.

## Results

### Ethical Clearance

The study was scientifically scrutinized and endorsed by the MMRU’s Scientific Review Board on June 16, 2021, and was granted scientific and ethical clearance by the Sierra Leone Ethics and Scientific Review Committee, Freetown, on July 5, 2021.

### Funding

Major contributions (salaries, accommodations, and consumables related to routine care) will be made as in-kind contributions by the participating centers. In August 2021, Dental Health International Netherlands approved the funding of the budgeted costs of the study (Table 2) after endorsing the study protocol. The Center of Tropical Medicine and Travel Medicine (of whom MPG is the head) serves as a sponsor of the study and serves as the guarantor for the funding (up to the sum described in the budget; Table 2), so that the study can be initiated right after approval.

**Table 2:** Budget calculation (includes both study sites).

Budget items			Cost (€)^a,b^
Personal protective equipment			1167.70
Data collection materials			114.80
Reimbursement follow-up visits			1600
**Additional fees**			
	Sierra Leonean ethical approval (students)	161.35	
	Open-access publication fees^c^	1500	
**In-kind contributions**			
	Microsoft Access and SPSS (IBM Corporation)^d^	N/A^e^	
	Personnel from Masanga Hospital and Lion Heart Medical Centre	N/A	

^a^A currency exchange rate of €1=US $1.14 is applicable.

^b^The total cost was €4543.85 (plus 10% overhead: €4998.24).

^c^There might be open access publication costs for the manuscript; hence, this cost factor was included in the calculation. The Center of Tropical Medicine and Travel Medicine, Amsterdam University Medical Center, serves as guarantor for eventual costs not being covered otherwise.

^d^These contributions were provided by the Masanga Medical Research Unit.

^e^N/A: not appliable.

### Data Collection

The projected start date of data collection was September 4, 2021, and data collection is expected to be complete in November 2022. A timeline is presented in Table 3.

**Table 3:** Timeline.

Task	December 2020 to April 2021	May 2021	June 2021	September 2021 to August 2022	September to November 2022	December 2022	January 2023	February 2023
Study protocol, CRF^a^, IC^b^ form, and PIF^c^	✓^d^	✓						
Funding	✓	✓						
Scientific Research Committee		✓	✓					
Protocol revision			✓					
Sierra Leone ethical council			✓					
Data collection				✓				
Data collection during follow-up contact				✓	✓			
Data analysis					✓			
Present data to local authorities						✓	✓	
Paper writing							✓	✓

^a^CRF: case record form.

^b^IC: informed consent.

^c^PIF: patient information folder.

^d^A checkmark indicates that the task will be performed during the specified period.

### Reporting of Study Results

Study results will be reported in accordance with the STROBE (Strengthening the Reporting of Observational Studies in Epidemiology) guidelines [18] for the reporting of prospective cohort studies. Study results are planned to be presented at the community level and during scientific meetings. One or several papers intended for publication in peer-reviewed journals will be prepared timely.

## Discussion

As only insufficient data are available in literature databases concerning the prevalence, characteristics, and management of complicated dental abscesses in Sierra Leone, our study aims to provide further information to improve treatment strategies.

## Appendix

Multimedia Appendix 1 Case record form.

Multimedia Appendix 2 Patient information folder.

Multimedia Appendix 3 Informed consent form.

Multimedia Appendix 4 Peer-review reports from the Masanga Medical Research Unit (MMRU).

## Abbreviations

AVPU: Alert, Responsive to Verbal Stimulus, Responsive to Pain Stimulus, Unresponsive
DELAY: Dental Abscess Study
DHIN: Dental Health International Netherlands
DNSI: Deep Neck Space Infection
LHMC: Lion Heart Medical Centre
MMRU: Masanga Medical Research Unit
PIN: Participant Identification Number
STROBE: Strengthening the Reporting of Observational Studies in Epidemiology
