# Supplementary material for: Complicated Odontogenic Infections at 2 District Hospitals in Tonkolili District, Sierra Leone: Protocol for a Prospective Observational Cohort Study (DELAY)
Source: JMIR Res Protoc. 2021 Dec 13;10(12):e33677. doi: 10.2196/33677 (PMC8713131; doi:10.2196/33677)
Supplement: Multimedia Appendix 1 [file resprot_v10i12e33677_app1.docx]

**CASE RECORD FORM**

DEntaL Abscess studY (DELAY): Prospective observational cohort study of complicated odontogenic infections in two district hospitals in Tonkolili District, Sierra Leone

**DESIGN OF THIS CASE RECORD FORM (CRF)**

This CRF has 3 modules:

**Module 1** to be filled out at the first day of admission to the health centre and on any change in clinical condition or change in treatment, and to be completed on the last day of admission to the health centre.
Table 1. (see Annex 1.) should be filled out on admission, on postoperative days 1, 2 and 7, and upon discharge.

**Module 2** to be completed at the first follow-up visit (approximately 4 weeks)

**Module 3** to be completed at the second follow-up visit (approximately 12 weeks)

**GENERAL GUIDANCE**

- The CRF is designed to collect data by interview, examination of the patient and use of the participants’ patient files including surgical reports
- By combining the site code with sequential participant numeration Participant Identification Numbers (PIN) will be obtained
- The Participant Identification Number and the date should be noted on each page
- Every question has to be answered. Leave sequential questions marked ‘If yes,…’ blank when they do not apply (i.e. when the answer is not yes)
- Please write clearly in ink, using BLOCK-CAPITAL LETTERS
- Place an (X) in the boxes to mark(tick) the answer. If an error is made, please strike through (--------) the data that should be deleted, and write the correct data aside it
- Mark unknown for any data that are not available or unknown
- Please try to avoid recording data outside of the dedicated areas
- When the duration of time is asked, please specify the amount of days, weeks or months and circle the correct value (either days, weeks or months) and strike through the answers not applying (--------)
- Medical photo’s should be taken upon admission and upon discharge and on each follow-up visit
- Please upload the medical photos on the research computer and external harddrive, stating the PIN of the participant
- Please keep all of the sheets for a single participant together e.g. with a staple
- Please check before discharging patient if the CRF is filled out completely and the medical photo’s at admission and at discharge are taken and send to the researchers

**MODULE 1: Complete on admission/enrolment**

To be filled out by CHO/MO/MMRU (as complete as possible) for linking questionnaire and microbial results:

Participant Identification Number:…………………………

Study site:..…………………………………………………………….

Date:………………………………………………………………………

Name of interviewer:………………………………………………

**(SOCIO)DEMOGRAPHICS**

1. Date : ………………………..DD/MM/YYYY /☐ Unknown
2. Age of Patient : ………………………………………………./☐ Unknown
3. Sex Patient : Male/Female /☐ Unknown
4. Marital status (circle correct answer) : Single/Married/Divorced/Widowed/Other

4b. If other, specify : ………………………………………………./☐ Unknown

1. Ethnicity : ………………………………………………./☐ Unknown
2. Place of Residence (city/village) : ………………………………………………./☐ Unknown
3. Highest level of education : ☐ No education

(Mark one box only) ☐ Some primary school

☐ Completed primary school

☐ Some secondary school

☐ Completed secondary school

☐ More than secondary school

☐ Unknown

1. Main occupation : ☐ Farming, Fishing

(Mark one box only) ☐ Mining

☐ Housewife

☐ Business, trader

☐ Health worker

☐ Student/pupil

☐ Retired

☐ Unemployed

☐ Other (specify): ………………………………………

☐ Unknown

1. Water availability : ☐ River or Pond / ☐ Unprotected dug well /

(Mark one box only) ☐ Rainwater tanker/cart/bottle / ☐ Borehole or tube well / ☐ Public tap / ☐ Piped water into dwelling/yard/plot/

☐ Other: …………………………………………………………………………..

☐ Unknown

1. Number of household members: ☐1/ ☐ 2/ ☐3/ ☐4/ ☐5/ ☐ 6/ ☐7/ ☐ 8/ ☐9/

(Mark one box only) ☐10/ ☐ >10 / ☐ Unknown

1. Comorbidities: ☐ Yes / ☐ No / ☐ Unknown

**PAST MEDICAL HISTORY**

11b. If yes, specify: ☐ HIV / ☐ TB / ☐ Sickle cell disease/ ☐ Diabetes Mellitus (DM)/

(Multiple choices possible) ☐ Obesity (BMI ≥ 30) / ☐ (suspected) malignancy / ☐ Psychiatric disorder / ☐ Substance abuse / ☐ Other, specify: …..……………………..

……………………………………………………………………………………………………………………………………………..…

…………………………………………………………………………………………………………………………./ ☐ Unknown

1. Any current medication use (with attention to antibiotics/diclofenac/ibuprofen/metamizol): ☐ Yes / ☐ No / ☐ Unknown

12b. If yes, specify type, dose, duration and administration-route:…………………………….…………………

………………………………………………………………………………………………………………………………………………..………………………………………………………………………………………………………………………………………………..

………………………………………………………………………………………………………………………………………………..

………………………………………………………………………………………………………………………………………………..

…………………………………………………………………………………………………………………………./ ☐ Unknown

1. Prior antibiotic treatment for current dental problem : ☐ Yes / ☐ No / ☐ Unknown

13b. If yes, which treatment (name of drug, administration-route, dose and duration):

(Multiple choices possible)

☐ Ceftriaxon … mg / ☐ OD ☐ BD ☐ TDS ☐ QDS / ☐ IV ☐ IM ☐ PO / duration in days: ….

☐ Ciprofloxacin … mg / ☐ OD ☐ BD ☐ TDS ☐ QDS / ☐ IV ☐ IM ☐ PO / duration in days: ….

☐ Amoxicillin … mg / ☐ OD ☐ BD ☐ TDS ☐ QDS / ☐ IV ☐ IM ☐ PO / duration in days: ….

☐ Ampicillin … mg / ☐ OD ☐ BD ☐ TDS ☐ QDS / ☐ IV ☐ IM ☐ PO / duration in days: ….☐ Cotrimoxazol … mg / ☐ OD ☐ BD ☐ TDS ☐ QDS / ☐ IV ☐ IM ☐ PO / duration in days: ….

☐ Ampliclox … mg / ☐ OD ☐ BD ☐ TDS ☐ QDS / ☐ IV ☐ IM ☐ PO / duration in days: ….

☐ Gentamicin … mg / ☐ OD ☐ BD ☐ TDS ☐ QDS / ☐ IV ☐ IM ☐ PO / duration in days: ….

☐ Doxycyclin … mg / ☐ OD ☐ BD ☐ TDS ☐ QDS / ☐ IV ☐ IM ☐ PO / duration in days: ….

☐ Other, please specify: ………………………………………………………………………………………………………………

……………………………..…………………………………………………………………………………………………………………`…………………………………………………………………………………………………………………………/ ☐ Unknown

1. Possession of a prescription for current dental problem : ☐ Yes / ☐ No / ☐ Unknown

14b. If yes, which treatment………………………………..………………..…………………………………….………………..

…………………………………………………………………………………………………………………………./ ☐ Unknown

1. Prior hospital admittance for the same problem : ☐ Yes / ☐ No / ☐ Unknown

15b. If yes, location and date of first admittance, duration of admittance (in days), (surgical and medicinal) treatment given during admittance: .……………………………………………………………………………

…………………………………………………………………………………………………………………………………………….....……………………………………………………………………………………………………………………………………………..…

……………………………………………………………………………………………………………………………………………..…

…………………………………………………………………………………………………………………………./ ☐ Unknown

1. Allergies : ☐ Yes / ☐ No known allergies / ☐ Unknown

16b. If yes, specify what for and reaction : ……………………………………………../ ☐ Unknown

1. Intoxications : Alcohol: ☐ Yes / ☐ No / ☐ Unknown

Smoking: ☐ Yes / ☐ No / ☐ Unknown

Other: ☐ Yes / ☐ No

Please specify if other: ………………………………..

1. Date of admission : ………………………………..DD/MM/YYYY / ☐ Unknown

**CLINICAL QUESTIONS**

1. First experienced complaint: ☐ Toothache

(Mark one box only) ☐ Jaw swelling/pain

☐ Trismus (inability to open mouth)

☐ Fever

☐ Generalized body pain/muscle ache

☐ Shortness of breath

☐ Other, please specify:………………………………………….

☐ Unknown

1. Time since start first complaints : … days/weeks/months (fill out and circle frequency)/

☐ Unknown

1. Site of origin of infection : ☐ Left lower jaw ☐ Right lower jaw

(Mark one box only and specify ☐ Left upper jaw ☐ Right upper jaw

Left or Right by circling, if appli- ☐ Neck ☐ Chest

cable) ☐ Above eye (L/R) ☐ Behind ear (L/R)

☐ Other, please specify:………………………………………….

☐ Unknown

Any of the following symptoms:

1. Toothache : ☐ Yes / ☐ No / ☐ Unknown

22b. if yes, duration of tootache (in days) : ………….……days / ☐ Unknown

1. Headache : ☐ Yes / ☐ No / ☐ Unknown

23b. if yes, duration of headache (in days) : ……….………days / ☐ Unknown

1. Earache : ☐ Yes / ☐ No / ☐ Unknown

24b. if yes, duration of earache (in days) : ……….………days / ☐ Unknown

1. Sleep disturbance : ☐ Yes / ☐ No / ☐ Unknown

25b. if yes, duration of insomnia (in days) : ……….………days / ☐ Unknown

1. Fever : ☐ Yes / ☐ No / ☐ Unknown

26b. if yes, duration of fever (in days) : ……….………days / ☐ Unknown

1. Swollen jaw : ☐ Yes / ☐ No / ☐ Unknown

27b. if yes, left or right side : ☐ Left lower / ☐ Right lower / ☐ Left upper / ☐ Right upper /

☐ Unknown

27c. if yes, duration of swollen jaw (in days) : ……….…..…days / ☐ Unknown

1. Swollen neck : ☐ Yes / ☐ No / ☐ Unknown

28b. if yes, duration of swollen neck (in days) : ……….………days / ☐ Unknown

1. Spontaneous discharge/pus : ☐ Yes / ☐ No / ☐ Unknown

29b. If yes, intra-orally or extra-orally : ☐ Extra-orally / ☐ Intra-orally /

☐ Unknown

29c. if yes, duration of pus discharge (in days) : ……….……..days / ☐ Unknown

1. Difficulty breathing (dyspnoea) : ☐ Yes / ☐ No / ☐ Unknown

30b. if yes, duration of dyspnoea (in days) : ……….………days / ☐ Unknown

1. Difficulty swallowing (dysphagia) : ☐ Yes / ☐ No / ☐ Unknown

31b. If yes, duration of dysphagia (in days) : ……….………days / ☐ Unknown

1. Difficulty opening mouth (trismus) : ☐ Yes / ☐ No / ☐ Unknown

32b. If yes, duration of trismus (in days) : ……….………days / ☐ Unknown

1. Stridor : ☐ Yes / ☐ No / ☐ Unknown

33b. If yes, duration of stridor (in days) : ……….………days / ☐ Unknown

1. Weight loss : ☐ Yes / ☐ No / ☐ Unknown

34b. If yes, how many kilograms (estimate) : ………………...KG / ☐ Unknown

1. Generalized body pain : ☐ Yes / ☐ No / ☐ Unknown

35b. if yes, duration of generalized body pain (in days): ……….………days / ☐ Unknown

1. Other complaints not specified above : ☐ Yes / ☐ No / ☐ Unknown

36b. If yes, specify : ………………………………………………………………………………………………

1. Predisposing factors : ☐ Dental caries suspected (rotten tooth)

(Multiple choices possible) ☐ Post extraction (after pulling of tooth)

**HEALTH BEHAVIOUR**

☐ None of the above ☐ Unknown

1. Consultation of traditional healer: ☐ Yes / ☐ No (if no->move to question 39)/ ☐ Unknown

38b. If yes, reason for seeking help of traditional healer : ……………………………………………

…………………………………………………………………………………………………………………………./ ☐ Unknown

38c. If yes, treatment received from traditional healer :

(Multiple choices possible) ☐ Pulling of tooth/teeth

☐ Application of herbs inside the mouth ☐ Application of boiled water inside the mouth

☐ Ingestion of herbs ☐ (Ingestion of) smoke

☐ Skin cuts ☐ Unknown

☐ Other, specify: ....……………………………………………………………………………………………………………… …….

38d. If yes, duration of treatment from traditional healer : …… days / ☐ Unknown

38e. If yes, reason for seeking help of traditional healer first:

(Multiple choices possible) ☐ Less costs ☐ For spiritual guidance

☐ Traditional healer is closer by in distance to access

☐ More faith in traditional healer than in hospital care

☐ Forced by relatives ☐ Unknown

☐ Other, specify: …………………………………………..…………….

………………………………………………………………………………………………………………………………………………

1. Reason for seeking care at healthcare facility now: ☐ Problem not resolved yet

(Multiple choices possible) ☐ Problem getting worse

☐ More faith in hospital care

☐ Unknown

☐ Other, specify: ……………………….

………………………………………………………………………………………………………………………………………………

1. Reason for delay seeking hospital care: ☐ No delay in seeking hospital care

(Multiple choices possible) ☐ Financial ☐ Transport

☐ Had to work ☐ Taking care of relatives

☐ Partner/family not agreeing in seeking care

☐ More faith in care by traditional healer

☐ Unknown

☐ Other, specify: ……………………………………………………

………………………………………………………………………………………………………………………………………………..

1. Beliefs about reason for infection : …………………………………………………………………….

……………………………………………………………………………………………………………………….. / ☐ Unknown

1. Use of toothbrush: ☐ Yes / ☐ No / ☐ Unknown

42b. If yes, how often: ..... per day/week/month (fill out and circle frequency)/ ☐ Unknown

42c. If yes, sharing of toothbrush : ☐ Yes / ☐ No / ☐ Unknown

42d. If sharing, with how many people: ☐1/ ☐ 2/ ☐3/ ☐4/ ☐5/ ☐ 6/ ☐7/ ☐ 8/ ☐9/ ☐10/

(Mark one box only) ☐ Unknown

42e. If sharing toothbrush, with whom: ☐ Parent(s) / ☐ Siblings / ☐ Grandparent(s) /

(Multiple choices possible) ☐ Other household members / ☐ Neighbours /

☐ At school / ☐ Community / ☐ Unknown /

☐ Other, specify: …………………………………………………..

1. Use of toothpaste : ☐ Yes / ☐ No / ☐ Unknown

43b. If yes, specify brand : ……………………………………………………………………... / ☐ Unknown

1. Use of commercial tooth picks : ☐ Yes / ☐ No / ☐ Unknown
2. Use of other material to clean teeth : ☐ Ash / ☐ Scrubbing with salt/

(Multiple choices possible) ☐ Eating bones / ☐ Country sticks /

☐ None of the above / ☐ Unknown /

☐ Other, specify: ……………………………………….

1. Diet : ☐ Staple only / ☐ Staple&Greens / ☐ Staple, greens & protein/

(Multiple choices possible) ☐ Vegetables / ☐ Fruits

1. Soda(sweet drink) consumption: .. a day/week/month (fill out&circle frequency)/☐Unknown

**CLINICAL EXAMINATION ON ADMISSION**

Vital signs on admission:

48. Airway : ☐ Open / ☐ Stridor / ☐ Obstructed / ☐ Unknown

49. Respiratory rate : ……………………………………… Per minute / ☐ Unknown

50. O^2^ saturation (without oxygen supply) : .…………………………… % / ☐ Unknown

50b. If oxygen supply necessary, O^2^ saturation with oxygen: ... % with .. L O2 / ☐ Unknown

1. Heart rate : ………………………………………. Per minute / ☐ Unknown
2. Blood pressure : ……………………………………………... mmHg / ☐ Unknown
3. Signs of shock : ☐ Cold extremities ☐ Weak pulse ☐ Restless or confused/

☐ Unknown

1. Temperature : ………………………………… Degrees Celsius / ☐ Unknown
2. AVPU-score : ☐ Alert/ ☐ Verbal/ ☐ Pain/ ☐ Unresponsive/ ☐ Unknown
3. Body measurements : Weight (in kilograms) : …………………… / ☐ Unknown

Length (in centimeters) : …………………… / ☐ Unknown

Clinical image - Medical photo taken: ☐ Yes / ☐ No (-> If no, take photo now now)

1. Inter-incisal opening to assess trismus, in cm : …………………………… / ☐ Unknown

(see Annex 1)

1. Interlobar distance, in cm (see Annex 1) : …………………………… / ☐ Unknown
2. Adam’s distance, in cm (see Annex 1) : …………………………... / ☐ Unknown

**CLINICAL EXAMINATION ON ADMISSION – TO BE FILLED OUT BY MEDICAL DOCTOR**

Filled out by Doctor/SACHO: ……..…………………………………………………………………………(name)

1. Clinically suspected for : ☐ Ludwig’s Angina

(Multiple choices possible) ☐ Cervical Necrotizing Fasciitis

☐ Suspicion of descending mediastinitis

☐ Pneumonia

☐ Thoracic empyema

☐ Suspicion of osteomyelitis

☐ Suspicion of meningitis/encephalitis/brain abscess

☐ Other, please specify:…………………………………………………..

☐ Unknown

1. Tooth suspected of infection : ………………………………………………………/ ☐ Unknown

(See numeration system below)


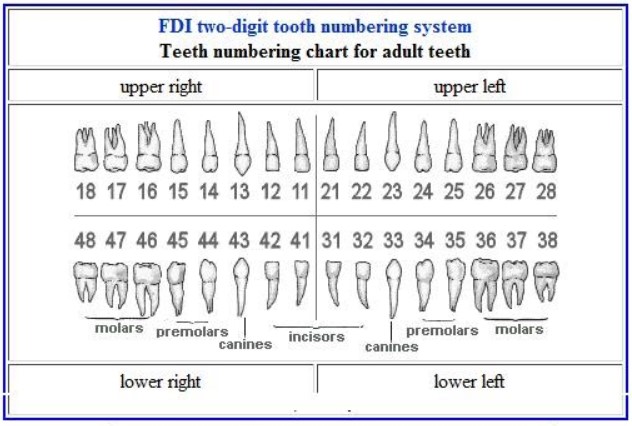
**Adults Children**


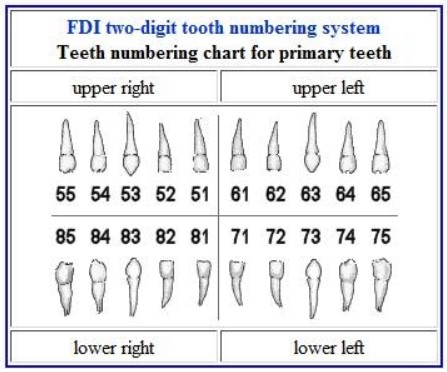


1. Amount of current teeth : ……………………………………………………. / ☐ Unknown
2. Caries : ☐ Yes / ☐ No / ☐ Unknown

63b. If yes, amount of caries : ☐1/ ☐ 2/ ☐3/ ☐4/ ☐5/ ☐ 6/ ☐7/ ☐ 8/ ☐9/ ☐10/ ☐>10 / ☐Unknown

63c. If yes, extensiveness : ☐ Superficial / ☐ Profundal / ☐ Unknown

1. Pus drainage in mouth cavity : ☐ Yes / ☐ No / ☐ Unknown
2. Pus drainage extra-orally : ☐ Yes / ☐ No / ☐ Unknown
3. Abscess : ☐ Yes / ☐ No / ☐ Unknown

66b. If no, induration : ☐ Yes / ☐ No / ☐ Unknown

1. Location of infection/swelling : ☐ Left lower jaw ☐ Right lower jaw

(Multiple choices possible) ☐ Left upper jaw ☐ Right upper jaw

☐ Submandibular ☐ Pharyngeal

☐ Pre-auricular (L/R) ☐ Retro-auricular (L/R)

☐ Temporal ☐ Above eye (L/R)

☐ Neck ☐ Chest

☐ Other, please specify:………………………………………….

☐ Unknown

1. Cervical lymphadenopathy : ☐ Yes / ☐ No / ☐ Unknown

☐ Not able to assess due to severe swelling

1. Soft tissue crepitations : ☐ Yes / ☐ No / ☐ Unknown

69b. if yes, location of crepitations : …………………………………………………….……./ ☐ Unknown

1. Pulmonary examination : …………………………………………………………………………………

………………………………………………………………………………………………………………………………………………..

1. Cardiovascular examination : …………………………………………………………………..

Including cardiac murmur on arrival : ☐ Yes / ☐ No / ☐ Unknown

71b. If yes, type of murmur : ☐ Systolic / ☐ Diastolic / ☐ Unknown

1. Suspicion of neurologic complications : ☐ Meningitis / ☐ Encephalitis /

(Multiple choices possible) ☐ Facialis paresis / ☐ Unknown /

☐ Other, specify…………………………………………

………………………………………………………………………………………………………………………………………………..

………………………………………………………………………………………………………………………………………………..

………………………………………………………………………………………………………………………………………………..

………………………………………………………………………………………………………………………………………………..

1. Haemoglobin (Hb) on admission : …………………… (in g/dL) / ☐ Unknown

**DIAGNOSTIC TESTS**

1. Platelets count : ………………… X 10^4^ / ☐ Unknown
2. Leukocyte count : ………………… X 10^9^ / ☐ Unknown
3. Leukocyte differentiation : ☐ Performed / ☐ Not performed / ☐ Unknown

☐ Neutrophils …… X 10^9^ / ☐ Unknown

☐ Lymphocytes …… X 10^9^ / ☐ Unknown

☐ Monocytes …… X 10^9^ / ☐ Unknown

☐ Eosinophils …… X 10^9^ / ☐ Unknown

☐ Basophils …… X 10^9^ / ☐ Unknown

1. Malaria on admission (mRDT) :☐ Positive / ☐ Negative / ☐ Unknown / ☐ Not performed
2. Malaria parasite slide :☐ Positive / ☐ Negative / ☐ Unknown / ☐ Not performed
3. HbsAg :☐ Positive / ☐ Negative / ☐ Unknown / ☐ Not performed
4. RVD/RVS :☐ Positive / ☐ Negative / ☐ Unknown / ☐ Not performed

80b. If yes, currently on ART : ☐ Yes / ☐ No / ☐ Unknown

80c. If yes, currently on cotrimoxazol : ☐ Yes / ☐ No / ☐ Unknown

1. Radiographic imaging performed : ☐ Yes / ☐ No / ☐ Unknown

81b If yes, specify : ☐ X-ray / ☐ Ultrasonography/ ☐ Unknown

81c. If yes, specify result : ………………………………………………………...………….

………………………………………………………………………………………………………………………………………………………

………………………………………………………………………………………………………………………………………………………

………………………………………………………………………………………………………………………………………………………

………………………………………………………………………………………………………………………………………………………

………………………………………………………………………………………………………………………………………………………

**TREATMENT**

1. Initial antibiotic treatment (specify type, dose, route of administration and duration)

Type Dosage Route of administration Duration

☐ Ceftriaxon … G / ☐ OD ☐ BD ☐ TDS ☐ QDS / ☐ IV ☐ IM ☐ PO / duration in days: …..

☐ Metronidazol … mg / ☐ OD ☐ BD ☐ TDS ☐ QDS / ☐ IV ☐ IM ☐ PO / duration in days: ….

☐ Gentamicin … mg / ☐ OD ☐ BD ☐ TDS ☐ QDS / ☐ IV ☐ IM ☐ PO / duration in days: ….

☐ Cotrimoxazol … mg / ☐ OD ☐ BD ☐ TDS ☐ QDS / ☐ IV ☐ IM ☐ PO / duration in days: ….

☐ Ciprofloxacin … mg / ☐ OD ☐ BD ☐ TDS ☐ QDS / ☐ IV ☐ IM ☐ PO / duration in days: ….

☐ Amoxicillin … mg / ☐ OD ☐ BD ☐ TDS ☐ QDS / ☐ IV ☐ IM ☐ PO / duration in days: ….

☐ Ampicillin … mg / ☐ OD ☐ BD ☐ TDS ☐ QDS / ☐ IV ☐ IM ☐ PO / duration in days: ….

☐ Ampliclox … mg / ☐ OD ☐ BD ☐ TDS ☐ QDS / ☐ IV ☐ IM ☐ PO / duration in days: ….

☐ Doxycyclin … mg / ☐ OD ☐ BD ☐ TDS ☐ QDS / ☐ IV ☐ IM ☐ PO / duration in days: ….

☐ Unknown

☐ If other, please specify : …………………………………………………………………………………

………………………………………………………………………………………………………………………………………………..

………………………………………………………………………………………………………………………………………………..

1. Initial other treatment : …………………………………………………………………………………

………………………………………………………………………………………………………………………………………………..

………………………………………………………………………………………………………………………………………………..

………………………………………………………………………………………………………………………… / ☐ Unknown

1. Surgical intervention : ☐ Yes / ☐ No / ☐ Unknown

84b. If yes, date of surgery : ………………………………..… DD/MM/YYYY / ☐ Unknown

84c. If yes, type of surgery : ☐ Incision and drainage, location: (Multiple choices possible) ☐ Pre-auricular

☐ Retro-auricular

☐ Submandibular

☐ Temporal

☐ Parietal

☐ Neck

☐ Chest

☐ Intra-oral

☐ Other: …..………………………………….

☐ Debridement

☐ Fasciectomy, location …..……………………………………

☐ Tracheostomy/cricothyrotomy

☐ Other: …..……………………………………………………………

☐ Unknown

84d. Type of analgesia : ☐ General / ☐ Local analgesia / ☐ Unknown

84e. Perioperative antibiotics : ☐ Yes / ☐ No / ☐ Unknown

If yes, specify type, dose, duration and administration-route : ……………………………………………….

………………………………………………………………………………………………………………………………………………..

………………………………………………………………………………………………………………… / ☐ Unknown

84f. If tracheostomy, duration in days : ……………………………………. / ☐ Unknown

84g. Same surgery teeth extraction : ☐ Yes / ☐ No / ☐ Unknown

1. Tooth extraction needed : ☐ Yes / ☐ No / ☐ Unknown

85b. If yes, which tooth/teeth (see images page 6) : ………………………………………………………..

………………………………………………………………………………………………………………… / ☐ Unknown

85c. Date of tooth extraction : ………………………….. DD/MM/YYYY / ☐ Unknown

1. Surgical revision : ☐ Yes / ☐ No / ☐ Unknown

86b. If yes, date of 2^nd^ surgery : ………………………….. DD/MM/YYYY / ☐ Unknown

86c. If yes, which procedure : …………………………………………………. / ☐ Unknown

86d. If yes, date of 3^rd^ surgery : ………………………….. DD/MM/YYYY / ☐ Unknown

86e. If yes, which procedure : …………………………………………………. / ☐ Unknown

86f. If yes, date of 4^th^ surgery : ………………………….. DD/MM/YYYY / ☐ Unknown

86g. If yes, which procedure : …………………………………………………. / ☐ Unknown

86h. If yes, date of 5^th^ surgery : ………………………….. DD/MM/YYYY / ☐ Unknown

86i. If yes, which procedure : …………………………………………………. / ☐ Unknown

1. Other noteworthy events during admission not mentioned previously (e.g. episode of deterioration, change in treatment strategy, other antibiotics/treatment prescribed etc):

………………………………………………………………………………………………………………………………………………………

………………………………………………………………………………………………………………………………………………………………………………………………………………………………………………………………………………………………………………

………………………………………………………………………………………………………………………………………………………

………………………………………………………………………………………………………………… ☐ None / ☐ Unknown

**SPECIMEN COLLECTION**

1. Pus sample taken : ☐ Yes, pus swab / ☐ Yes, pure pus / ☐ No / ☐ Unknown

(Multiple choices possible -> if answer is no, consult with PI about necessity to take sample)

88b. If yes, date of pus collection : ……………………………….. DD/MM/YYYY / ☐ Unknown

88c. If yes, timing of pus collection : ☐ Upon admission / ☐ Perioperatively /

☐ Upon discharge / ☐ Unknown

1. Discharge : ☐ With medical agreement / ☐ Against medical advice / ☐ Unknown

**DISCHARGE DATA**

89b. If against medical advice, state reason why : ……………………………………………………………...

……………………………………………………………………………………………………………………….…/ ☐ Unknown

1. Discharge date : ……………………………………………………….……DD/MM/YYYY / ☐ Unknown
2. Duration of admission : ............................................................. in days / ☐ Unknown
3. Overview of antibiotics received during stay at hospital: ………………………………………………………..

………………………………………………………………………………………………………………………………………………………

………………………………………………………………………………………………………………………………………………………

………………………………………………………………………………………………………………………………………………………

………………………………………………………………………………………………………………………………………………………

………………………………………………………………………………………………………………………………………………………

………………………………………………………………………………………………………………………………………………………

………………………………………………………………………………………………………………………………………………………

………………………………………………………………………………………………………………………………./ ☐ Unknown

1. Any other comorbidities diagnosed during admission:

(Multiple choices possible) ☐ HIV ☐ Hypertension

☐ TB ☐ Diabetes mellitus

☐ Sickle cell disease ☐ Anaemia

☐ Malaria

☐ Other, specify: …………………………………………………….

☐ Unknown

1. Medical photo taken upon discharge: ☐ Yes / ☐ No (-> If no, take photo now now)
2. Outcome : ☐ No remaining complaints

(Multiple choices possible) ☐ Wound not closed yet

☐ Skin grafted

☐ Skin graft necessary

☐ Pulling of teeth still needs to be done

☐ Facialis paresis L/R

☐ Lateralization L/R lower/upper extremity

☐ Unknown

☐ Other, ………………………………………………………………………………

……………………………………………………………………………………………………………………………………………….. ………………………………………………………………………………………………………………………………………………..

1. Mortality : ☐ Yes / ☐ No / ☐ Unknown

96b. If mortality yes, date of death : .…………………….. DD/MM/YYYY / ☐ Unknown

96c. If mortality yes, reported cause of death : ……………………………………………………….

………………………………………………………………………………………………………………………… / ☐ Unknown

**SCHEDULE FOR FIRST AND SECOND FOLLOW-UP CONTACT**

Date of first follow-up visit (4 weeks from now) : ……………………………….……………………....(DD/MM/YYYY)
Date of second follow-up visit (12 weeks from now):…………………………………………………..(DD/MM/YYYY)

-----------------------------------------------------END OF MODULE 1--------------------------------------------------------

**MODULE 2: Complete on follow-up visit 1**

**FOLLOW-UP VISIT 1 (after approximately 4 weeks after discharge from hospital)**

(If patient is no longer alive, please report on date of death and circumstances on page 13)

Current Date : ………………………………………………DD/MM/YYYY

**MEDICATION USE**

1. Medication use after discharge from hospital (with attention to antibiotics/diclofenac/ ibuprofen/metamizol):…………………………………………………………………………………………………………….
   …………………………………………………………………………….……………………………………….…../ ☐ Unknown

**CURRENT CLINICAL CONDITION FOLLOW-UP VISIT 1**

1. Current clinical condition as reported by patient (mark one box only):

☐ Completely recovered, no complaints

☐ Almost completely recovered, small complaints

☐ Not recovered at all, still many complaints

☐ Readmission necessary

☐ Recovered completely initially but developed complaints again

☐ Other, specify: ……………………………………………………………………………………………………………………

☐ Unknown

1. Any of the following symptoms remaining or reoccurring (multiple choices possible):

☐ Toothache ☐ Fever

☐ Dyspnoea ☐ Trismus

☐ Wound

☐ Swollen jaw; if yes, left or right side:

☐ Left lower /☐ Left upper / ☐ Right lower / ☐ Right upper / ☐ Unknown

☐ Discharge/pus; if yes, intra-orally or extra-orally:

☐ Extra-orally / ☐ Intra-orally / ☐ Unknown

☐ Other complaints not specified above : ……………………………………………………….

**HEALTH BEHAVIOUR**

………………………………………………………………………………………………………………………… / ☐ Unknown

1. Use of toothbrush **changed** after discharge from hospital : ☐ Yes / ☐ No / ☐ Unknown

4b. If yes, specify: ……………………………………………………………………………………………… / ☐ Unknown

4c. If yes, brushing how often now: ...... per day/week/month(circle frequency) / ☐ Unknown

4d. If yes, use of dental material : ☐ Toothpaste / ☐ Tooth picks /

☐ Other, specify: ………....…………… / ☐ Unknown

1. Consultation of any ‘therapist’ for tooth problem after discharge: ☐Yes/ ☐ No/ ☐Unknown

5b. Specify ‘therapist’: ☐ Traditional healer/ ☐ Dental worker / ☐ Employee in other hospital/ ☐ Unknown/ ☐ Other, specify: ………………………………………..

5c. If yes, reason for seeking help again : ……………………………………………………………………

………………………………………………………………………………………………………………………………/ ☐ Unknown

5d. Treatment received of the above mentioned ‘therapist’ :

☐ Application of herbs inside the mouth ☐ Application of boiled water inside the mouth

☐ Ingestion of herbs ☐ (Ingestion of) smoke

☐ Skin cuts ☐ Pulling of tooth/teeth

☐ Unknown ☐ Other, specify: ……………………………………………………

5e. Amount of teeth pulled: ☐0 / ☐1/ ☐ 2/ ☐3/ ☐4/ ☐5/ ☐ 6/ ☐7/ ☐ 8/ ☐9/ ☐10/ ☐ >10/

☐ Unknown

Vital signs on follow-up visit 1:

**CLINICAL EXAMINATION ON FOLLOW-UP VISIT 1**

6. Respiratory rate : ……………………………………… Per minute / ☐ Unknown

7. O^2^ saturation : .…………………………… % / ☐ Unknown

1. Heart rate : ………………………………………. Per minute / ☐ Unknown
2. Blood pressure : ……………………………………………... mmHg / ☐ Unknown
3. Temperature : ………………………………… Degrees Celsius / ☐ Unknown
4. Weight (in kilograms) : ….…………………… ……… / ☐ Unknown

Clinical image - Medical photo taken: ☐ Yes / ☐ No (-> If no, take photo now now)

1. Open wound remaining : ☐ Yes / ☐ No / ☐ Unknown

12b. If yes, location of open wound : ☐ Left lower jaw ☐ Right lower jaw

☐ Left upper jaw ☐ Right upper jaw

☐ Submandibular ☐ Neck

☐ Chest ☐ Pre-auricular (L/R)

☐ Retro-auricular (L/R) ☐ Above eye (L/R)

☐ Other, please specify:………………………………………….

☐ Unknown

1. Swelling remaining : ☐ Yes / ☐ No / ☐ Unknown

13b. If yes, location of infection/swelling: ☐ Left lower jaw ☐ Right lower jaw

☐ Left upper jaw ☐ Right upper jaw

☐ Submandibular ☐ Pharyngeal

☐ Pre-auricular (L/R) ☐ Retro-auricular (L/R)

☐ Temporal ☐ Above eye (L/R)

☐ Neck ☐ Chest

☐ Other, please specify:………………………………………….

☐ Unknown

1. Interlobar distance, in cm (see Annex 1) : ………………………………… / ☐Unknown
2. Inter-incisal distance, in cm (see Annex 1) : ………………………………… / ☐Unknown
3. Adam’s distance, in cm (see Annex 1) : ………………………………… / ☐Unknown
4. Number of current teeth : ……………………………………………………..…… / ☐Unknown
5. Amount of caries : ☐1/ ☐ 2/ ☐3/ ☐4/ ☐5/ ☐ 6/ ☐7/ ☐ 8/ ☐9/ ☐10/ ☐>10 / ☐Unknown
6. Pus drainage in mouth cavity : ☐ Yes / ☐ No / ☐ Unknown
7. Pus drainage extra-orally : ☐ Yes / ☐ No / ☐ Unknown
8. Abscess : ☐ Yes / ☐ No / ☐ Unknown

**TREATMENT**

1. Treatment follow-up visit 1. : ☐ No treatment necessary

☐ Admission for incision and drainage

☐ Admission for skin graft

☐ Admission for antibiotic treatment, specify antibiotics: ………………………………………………………………

☐ Outpatient treatment with antibiotics, specify antibiotics: ………………………………………………………………

☐ Tooth pulling by dental care worker as outpatient

☐ Follow-up visit 2. after 8 more weeks advised

☐ Other, please specify:………………………………………….

☐ Unknown

**OPEN FIELD**

1. Any important remaining information not mentioned above : ☐ None

………………………………………………………………………………………………………………………………………….…………………………………………………………………………………………………………………………………………………….

……………………………………………………………………………………………………………………………………………….

……………………………………………………………………………………………………………………………………………….

……………………………………………………………………………………………………………………………………………….

……………………………………………………………………………………………………………………………………………….

……………………………………………………………………………………………………………………………………………….

……………………………………………………………………………………………………………………………………………….

……………………………………………………………………………………………………………………………………………….

……………………………………………………………………………………………………………………………………………….

……………………………………………………………………………………………………………………………………………….

………………………………………………………………………………………………………………………………………………...………………………………………………………………………………………………………………………………………………

….……………………………………………………………………………………………………………………../ ☐ Unknown

**IN CASE PATIENT DIED AFTER DISCHARGE BEFORE FOLLOW-UP VISIT 1**

1. Date of death : ………………….…………………….. DD/MM/YYYY / ☐ Unknown

24b. Location of death: ☐ Home / ☐ Different hospital / ☐ Unknown / ☐ Other: ……………. ………………………………………………………………………………………………………………………………………………………
24c. Suspected reason for death : ………………………………………………………………………………..

………………………………………………………………………………………………………………………………………………………………………………………………………………………………………………………………………………………../ ☐ Unknown

**SCHEDULING FOLLOW-UP VISIT 2**

1. Date for second follow-up visit (8 weeks from now): …………………(DD/MM/YYYY)

**MODULE 3: Complete on follow-up visit 2**

**FOLLOW-UP CONTACT 2 (after approximately 12 weeks after discharge from hospital)**

(If patient is no longer alive, please report on date of death and circumstances on page 16)

Current Date : ………………………………………………DD/MM/YYYY

**MEDICATION USE**

1. Medication use after follow-up visit 1 (with attention to antibiotics/diclofenac/ ibuprofen/metamizol): ………………………………………………………………………………………..………………….
   …………………………………………………………………………….……………………………………….…../ ☐ Unknown

**CURRENT CLINICAL CONDITION FOLLOW-UP VISIT 2**

1. Current clinical condition as reported by patient (mark one box only):

☐ Completely recovered, no complaints

☐ Almost completely recovered, small complaints

☐ Not recovered at all, still many complaints

☐ Readmission necessary

☐ Recovered completely initially but developed complaints again

☐ Other, specify: ……………………………………………………………………………………………………………………

☐ Unknown

1. Any of the following symptoms remaining or reoccurring (multiple choices possible):

☐ Toothache ☐ Fever

☐ Dyspnoea ☐ Trismus

☐ Wound

☐ Swollen jaw; if yes, left or right side:

☐ Left lower /☐ Left upper / ☐ Right lower / ☐ Right upper / ☐ Unknown

☐ Discharge/pus; if yes, intra-orally or extra-orally:

☐ Extra-orally / ☐ Intra-orally / ☐ Unknown

☐ Other complaints not specified above : ……………………………………………………….

………………………………………………………………………………………………………………………… / ☐ Unknown

**HEALTH BEHAVIOUR**

1. Use of toothbrush **changed** after follow-up visit 1 : ☐ Yes / ☐ No / ☐ Unknown

4b. If yes, specify: ……………………………………………………………………………………………… / ☐ Unknown

4c. If yes, brushing how often now: ...... per day/week/month(circle frequency) / ☐ Unknown

4d. If yes, use of dental material : ☐ Toothpaste / ☐ Tooth picks /

☐ Other, specify: ………....…………… / ☐ Unknown

1. Consultation of any ‘therapist’ for tooth problem after last visit : ☐Yes/ ☐ No/ ☐Unknown

5b. Specify ‘therapist’: ☐ Traditional healer/ ☐ Dental worker / ☐ Employee in other hospital/ ☐ Unknown/ ☐ Other, specify: ………………………………………..

5c. If yes, reason for seeking help again : ……………………………………………………………………

………………………………………………………………………………………………………………………………/ ☐ Unknown

5d. Treatment received of the above mentioned ‘therapist’ :

☐ Application of herbs inside the mouth ☐ Application of boiled water inside the mouth

☐ Ingestion of herbs ☐ (Ingestion of) smoke

☐ Skin cuts ☐ Pulling of tooth/teeth

☐ Unknown ☐ Other, specify: ……………………………………………………

5e. Amount of teeth pulled: ☐0 / ☐1/ ☐ 2/ ☐3/ ☐4/ ☐5/ ☐ 6/ ☐7/ ☐ 8/ ☐9/ ☐10/ ☐ >10/

☐ Unknown

Vital signs on follow-up visit 2:

**CLINICAL EXAMINATION ON FOLLOW-UP VISIT 2**

1. Respiratory rate : ……………………………………… Per minute / ☐ Unknown
2. O^2^ saturation : .…………………………… % / ☐ Unknown
3. Heart rate : ………………………………………. Per minute / ☐ Unknown
4. Blood pressure : ……………………………………………... mmHg / ☐ Unknown
5. Temperature : ………………………………… Degrees Celsius / ☐ Unknown
6. Weight (in kilograms) : ….…………………… ……… / ☐ Unknown

Clinical image - Medical photo taken: ☐ Yes / ☐ No (-> If no, take photo now now)

1. Open wound remaining : ☐ Yes / ☐ No / ☐ Unknown

12b. If yes, location of open wound : ☐ Left lower jaw ☐ Right lower jaw

☐ Left upper jaw ☐ Right upper jaw

☐ Submandibular ☐ Neck

☐ Chest ☐ Pre-auricular (L/R)

☐ Retro-auricular (L/R) ☐ Above eye (L/R)

☐ Other, please specify:………………………………………….

☐ Unknown

1. Swelling remaining : ☐ Yes / ☐ No / ☐ Unknown

13b. If yes, location of infection/swelling: ☐ Left lower jaw ☐ Right lower jaw

☐ Left upper jaw ☐ Right upper jaw

☐ Submandibular ☐ Pharyngeal

☐ Pre-auricular (L/R) ☐ Retro-auricular (L/R)

☐ Temporal ☐ Above eye (L/R)

☐ Neck ☐ Chest

☐ Other, please specify:………………………………………….

☐ Unknown

1. Interlobar distance, in cm (see Annex 1) : ………………………………… / ☐Unknown
2. Inter-incisal distance, in cm (see Annex 1) : ………………………………… / ☐Unknown
3. Adam’s distance, in cm (see Annex 1) : ………………………………… / ☐Unknown
4. Number of current teeth : ……………………………………………………..…… / ☐Unknown
5. Amount of caries : ☐1/ ☐ 2/ ☐3/ ☐4/ ☐5/ ☐ 6/ ☐7/ ☐ 8/ ☐9/ ☐10/ ☐>10 / ☐Unknown
6. Pus drainage in mouth cavity : ☐ Yes / ☐ No / ☐ Unknown
7. Pus drainage extra-orally : ☐ Yes / ☐ No / ☐ Unknown
8. Abscess : ☐ Yes / ☐ No / ☐ Unknown

**TREATMENT**

1. Treatment follow-up visit 2. : ☐ No treatment necessary

☐ Admission for incision and drainage

☐ Admission for skin graft

☐ Admission for antibiotic treatment, specify antibiotics: ………………………………………………………………

☐ Outpatient treatment with antibiotics, specify antibiotics: ………………………………………………………………

☐ Tooth pulling by dental care worker as outpatient

☐ Other, please specify:………………………………………….

☐ Unknown

1. Any important remaining information not mentioned above : ☐ None

**OPEN FIELD**

………………………………………………………………………………………………………………………………………….…………………………………………………………………………………………………………………………………………………….

……………………………………………………………………………………………………………………………………………….

……………………………………………………………………………………………………………………………………………….

……………………………………………………………………………………………………………………………………………….

……………………………………………………………………………………………………………………………………………….

……………………………………………………………………………………………………………………………………………….

……………………………………………………………………………………………………………………………………………….

……………………………………………………………………………………………………………………………………………….

……………………………………………………………………………………………………………………………………………….

……………………………………………………………………………………………………………………………………………….

……………………………………………………………………………………………………………………………………………….

……………………………………………………………………………………………………………………………………………….

………………………………………………………………………………………………………………………../ ☐ Unknown

**IN CASE PATIENT DIED AFTER FOLLOW-UP VISIT 1 AND BEFORE FOLLOW-UP CONTACT 2**

1. Date of death : …………………………………………..………………(DD/MM/YYYY)/ ☐ Unknown

24b. Location of death: ☐ Home / ☐ Different hospital / ☐ Unknown / ☐ Other: ………..…..

………………………………………………………………………………………………………………………………../ ☐ Unknown
24c. Suspected reason for death : ………………………………………………………………………………...

………………………………………………………………………………………………………………………………………………………

..…………………………………………………………………………………………………………………………….../ ☐ Unknown

**END OF CRF**

Thank you for participating!

ANNEX 1.

Table 1.

| **Measurement** | **Anatomic site of measurement** | **When measured (specify date)** | **Outcome (in cm)** |
| --- | --- | --- | --- |
| Interlobar distance  Measured with tape measure | From tip of tragus, to gonion, to contralateral tip of tragus | - upon admission: |  |
|  |  | - postoperative   day 1: |  |
|  |  | - postoperative   day 2: |  |
|  |  | - postoperative   day 7: |  |
|  |  | - At discharge: |  |
|  |  | - Follow-up 1: |  |
|  |  | - Follow-up 2: |  |
| Maximal interincisal mouth-opening capacity/Trismus  Measured with Vernier caliper | From incisal edge of maxillary central incisor to incisal edge of mandibular central incisor | - upon admission: |  |
|  |  | - postoperative   day 1: |  |
|  |  | - postoperative   day 2: |  |
|  |  | - postoperative   day 7: |  |
|  |  | - At discharge: |  |
|  |  | - Follow-up 1: |  |
|  |  | - Follow-up 2: |  |
| Adam’s distance  Measured with tape measure | From the laryngeal cartilage to the Vermillion border of the lower lip at rest | - upon admission: |  |
|  |  | - postoperative   day 1: |  |
|  |  | - postoperative   day 2: |  |
|  |  | - postoperative   day 7: |  |
|  |  | - At discharge: |  |
|  |  | - Follow-up 1: |  |
|  |  | - Follow-up 2: |  |

ANNEX 2.

Laboratory results local lab:

Type of specimen and results: ………………………………………………………………………………………………..

………………………………………………………………………………………………………………………………………………..

………………………………………………………………………………………………………………………………………………..

………………………………………………………………………………………………………………………………………………..

………………………………………………………………………………………………………………………………………………..

………………………………………………………………………………………………………………………………………………..

………………………………………………………………………………………………………………………………………………..

………………………………………………………………………………………………………………………………………………..

………………………………………………………………………………………………………………………………………………..

………………………………………………………………………………………………………………………………………………..

………………………………………………………………………………………………………………………………………………..

………………………………………………………………………………………………………………………………………………..

………………………………………………………………………………………………………………………………………………..

………………………………………………………………………………………………………………………………………………..

………………………………………………………………………………………………………………………………………………..

………………………………………………………………………………………………………………………………………………..

………………………………………………………………………………………………………………………………………………..

………………………………………………………………………………………………………………………………………………..

………………………………………………………………………………………………………………………………………………..

………………………………………………………………………………………………………………………………………………..

………………………………………………………………………………………………………………………………………………..

………………………………………………………………………………………………………………………………………………..

………………………………………………………………………………………………………………………………………………..

………………………………………………………………………………………………………………………………………………..

………………………………………………………………………………………………………………………………………………..

………………………………………………………………………………………………………………………………………………..

………………………………………………………………………………………………………………………………………………..

………………………………………………………………………………………………………………………………………………..

………………………………………………………………………………………………………………………………………………..

………………………………………………………………………………………………………………………………………………..

………………………………………………………………………………………………………………………………………………..

………………………………………………………………………………………………………………………………………………..

………………………………………………………………………………………………………………………………………………..

………………………………………………………………………………………………………………………………………………..

………………………………………………………………………………………………………………………………………………..

………………………………………………………………………………………………………………………………………………..

………………………………………………………………………………………………………………………………………………..

………………………………………………………………………………………………………………………………………………..

………………………………………………………………………………………………………………………………………………..

………………………………………………………………………………………………………………………………………………..

………………………………………………………………………………………………………………………………………………..

………………………………………………………………………………………………………………………………………………..

………………………………………………………………………………………………………………………………………………..

Laboratory results Münster lab:

Type of specimen and results: ………………………………………………………………………………………………..

………………………………………………………………………………………………………………………………………………..

………………………………………………………………………………………………………………………………………………..

………………………………………………………………………………………………………………………………………………..

………………………………………………………………………………………………………………………………………………..

………………………………………………………………………………………………………………………………………………..

………………………………………………………………………………………………………………………………………………..

………………………………………………………………………………………………………………………………………………..

………………………………………………………………………………………………………………………………………………..

………………………………………………………………………………………………………………………………………………..

………………………………………………………………………………………………………………………………………………..

………………………………………………………………………………………………………………………………………………..

………………………………………………………………………………………………………………………………………………..

………………………………………………………………………………………………………………………………………………..

………………………………………………………………………………………………………………………………………………..

………………………………………………………………………………………………………………………………………………..

………………………………………………………………………………………………………………………………………………..

………………………………………………………………………………………………………………………………………………..

………………………………………………………………………………………………………………………………………………..

………………………………………………………………………………………………………………………………………………..

………………………………………………………………………………………………………………………………………………..

………………………………………………………………………………………………………………………………………………..

………………………………………………………………………………………………………………………………………………..

………………………………………………………………………………………………………………………………………………..

………………………………………………………………………………………………………………………………………………..

………………………………………………………………………………………………………………………………………………..

………………………………………………………………………………………………………………………………………………..

………………………………………………………………………………………………………………………………………………..

………………………………………………………………………………………………………………………………………………..

………………………………………………………………………………………………………………………………………………..

………………………………………………………………………………………………………………………………………………..

………………………………………………………………………………………………………………………………………………..

………………………………………………………………………………………………………………………………………………..

………………………………………………………………………………………………………………………………………………..

………………………………………………………………………………………………………………………………………………..

………………………………………………………………………………………………………………………………………………..

………………………………………………………………………………………………………………………………………………..

………………………………………………………………………………………………………………………………………………..

………………………………………………………………………………………………………………………………………………..

………………………………………………………………………………………………………………………………………………..

………………………………………………………………………………………………………………………………………………..

………………………………………………………………………………………………………………………………………………..

………………………………………………………………………………………………………………………………………………..
