# Supplementary material for: Complicated Odontogenic Infections at 2 District Hospitals in Tonkolili District, Sierra Leone: Protocol for a Prospective Observational Cohort Study (DELAY)
Source: JMIR Res Protoc. 2021 Dec 13;10(12):e33677. doi: 10.2196/33677 (PMC8713131; doi:10.2196/33677)
Supplement: Multimedia Appendix 2 [file resprot_v10i12e33677_app2.docx]

**Patient information folder about participating in medico-scientific research**

*Informal title:* “DELAY study: A study about the occurrence, patient-reported complaints, physical findings and effect of treatment for severe tooth infections in two district hospitals in Tonkolili District, Sierra Leone*”*

*Official title:* “DEntaL Abscess studY (DELAY): Prospective observational cohort study of complicated odontogenic infections in two district hospitals in Tonkolili District, Sierra Leone

**Introduction**

Dear Sir, Madam,

We are kindly requesting you to participate in medico-scientific research. Participation is on a voluntarily basis. To participate in the study, signed consent is necessary. This letter is given attention to you because you are diagnosed with a serious tooth infection by one of our employees.

Before you decide whether or not to participate in this study, you will receive information about the study. Read this information carefully (or ask someone to read this information out loud to you) and ask the researchers (who will be specified by name at the end of this document) for more information if you have any questions. It is also an option to ask the independent expert (named at the end of the document too) for further information. You can also discuss whether or not to participate with your partner, family or friends.

1. **General information**

This study has been developed by the Masanga Medical Research Unit of Masanga Hospital and will be coordinated by employees of Masanga Hospital (Masanga) and Lion Heart Medical Centre (Yele). Since the study is observational, there is no set number on how many participants will be asked to participate in the study. Participants will be asked to join the study between September 2021 and August 2022.

1. **Purpose of this study**

You have been diagnosed with a severe tooth infection. This means that you will need to be admitted to the hospital for treatment. When a tooth infection exists for a long time, progress of the infection can cause a lot of pain, swelling and an infection that requires treatment with antibiotics via a canula. Also surgery can be necessary to make sure the pus can come out. Currently it is not known how many people in Tonkolili District get these more severe tooth infections. Also, it is not known why some people get these more serious infections and others do not, what symptoms the majority of people are experiencing and with what kind of problems most people come to the hospital (for example, with difficulty eating). The purpose of this study is to get a better idea about these things, so that we can treat the tooth infection correctly. What is important to know is that the treatment that you receive will be the same whether you are participating in the study or not. If you choose to participate in the study, we will ask you questions about your personal information (for example, where do you live), about the complaints you have (for example, do you have a toothache and if yes for how long) and we will register information about the physical examination and treatment you receive. We will ask for a separate signature to allow the research to make and use a picture of your wound. All pictures will be kept highly confidentially and will only be used for research purposes. This includes publication of anonymized (non-identifiable) picture material. Also, a sample of pus from the infection site will be collected and checked to identify which bacteria are causing the infection. When you have been discharged, we will ask you to come back to the hospital for another check-up after four weeks and after 12 weeks (three months) for which financial reimbursement for transport costs/airtime is available.

1. **Background of the study**

Currently it has not been known who develops a serious tooth infection, which symptoms are experienced mostly and what the general findings are when physical examination is performed in rural areas like Tonkolili District. The bacteria’s causing severe tooth infections have not been identified yet. Moreover, the outcome after treatment during admission and after four and 12 weeks has not been registered previously. Because of this, at this moment it is difficult to give people tailored treatment or try to prevent serious tooth infections at community level. This study is developed to get a better view on factors influencing the occurrence of and progress to severe tooth infection and the effect of treatment.

1. **What it means to participate in the study**

If you participate in the study, you will be asked to answer a questionnaire (taken by a local research nurse) and you will undergo physical examination at three different moments, namely during admission and after four and 12 weeks approximately after discharge. Whether you participate in the study or not does not have any influence on the treatment you receive (so the treatment will be the same either way).

**Measurements**

When you are admitted and you agree to participate in the study, a researcher will come to you to fill out a questionnaire together with you. The physical examination findings will be registered upon and during admission, the treatment you receive during the admission will be registered too, and medical photo’s will be taken. If there is a swelling in the face, jaw or neck present, measurements of the size will be taken. This will be repeated if surgery is necessary, namely at day 1, day 2 and day 7 postoperatively, and upon discharge. It will also be repeated if you receive antibiotics only. If possible during admission, pus will be collected from the infection site, and it will be analysed to identify the bacteria’s in it. The treatment you receive will be copied from your patient file. A questionnaire and physical examination will be repeated during the follow-up visits four and 12 weeks after you have been discharged, as well as a medical photo.

1. **What is expected of you**

You will be asked to fill out the questionnaire together with the researcher when you are admitted to the hospital. You are asked to come back to the hospital four and 12 weeks after discharge to repeat the questionnaire and physical examination, and to hear how you are doing.

1. **Possible adverse effects**

As the research is strictly observational and we will treat you according to normal treatment protocol, no possible adverse effects are expected from participating in the study.

1. **Possible arguments in favour and against participating in the study**

It is important that you weigh the possible advantages and disadvantages before you decide to participate in the study or not. There is no direct benefit for you to participate in the study. However, your participation can be beneficial for the community as it can increase knowledge about severe tooth infections that require admission in the hospital. Possible disadvantage of participating in the study will be the time it takes to fill out the questionnaire and the time it takes to come back to the hospital for the follow-up visits.

1. **If you don’t want to participate in the study or if you wish to end your participation**

You yourself decide if you want to participate in the study or not. Participation is completely voluntary. If you do participate, you can change your mind at any moment and you can decide to stop participating at any moment. You don’t have to tell us why you want to stop. We do ask you to tell the researcher as soon as possible if you choose to stop participating. The data that is collected up until the moment you decide to stop, will be used for research purposes. If you wish so, the body fluid which is collected (the pus specimen) can be destroyed.

1. **End of the study**

Your participation to the study stops when:

- All investigations as described in paragraph 4 have taken place
- You choose to stop
- The end of the complete research period has been reached, when the study period is over. This is about 15 months after the start of the study
- Researchers agree it is better for you to stop participating
- Masanga Hospital/Lion Heart Medical Centre, the government or the Sierra Leone Ethics and Scientific Review Committee decide to stop the study

The study ends after the study period is finished (September 2021 – August 2022)

1. **Use and storage of your data and body fluids (pus specimen)**

For study purposes it is necessary that your body fluid (pus) and medical data are collected and analysed. Every participant gets an individual code which is registered on your participant forms and on the body fluid container. Your name and other personal data that could directly identify you will not be registered to provide anonymity.

**Your data**

All your data will be handled with care and will remain confidential. The research team only knows your Personal Identification Number (PIN). The key for the code will be known by the researchers only. In reports about the research, only the anonymous PIN will be used.

There are some people who are allowed to look into your personal and medical records. This is to make sure the study is executed conform certain standards and that it is reliable. They will keep your data confidential. If you sign the consent form, you agree upon gathering, storing, and analysing your medical and personal records.

The research unit will store your records for 15 years and subsequently destroy it.

**Your bodily fluids**

If pus has been collected during your admission, it will be stored at the laboratory site in Münster, Germany or at the Masanga Medical Research Unit (MMRU) for a period of 15 years and it can be rechecked at a later stage to ensure the quality and validity of the study.

1. **Insurance for study participants**

If you choose to participate in the study, you will not have any extra risks other than if you do not participate in the study. As such, an additional trial participant insurance of study participants is not provided.

1. **Financial reimbursement**

Since the follow-up visits after four and 12 weeks will consume transport costs and airtime, a reimbursement will be handed over to you after the follow-up visits have been finished (50.000 Leones after each visit). However, you will not be paid to participate in the study whatsoever.

1. **Any questions?**

If you have any questions, you are invited to ask the researchers for answers. For independent advice on participating in the study or not, you can ask the independent expert. He knows a lot about the study, but he is not involved in the study.

If you have any complaints about the study you can contact the coordinator of the study. You also have the right to contact the Sierra Leone Ethics and Scientific Research Committee if you have any issues with the study. Contact details are attached in **Annex A:** Contact Details.

1. **Signing Informed Consent**

When you have had adequate time to think about participating or not and you have been given the chance to ask the questions that you wanted to, you will be asked to decide to participate in the study. If you decide to participate in the study, we ask you to sign the informed consent form (**Annex 4**: **Informed consent form**) and to hand it over to the research team. By providing your informed consent you state that you have read and understood the information about the study and that you agree with participating in the study.

The signed informed consent form will be stored by the research team. A copy or a second informed consent form will be handed over to you for you to keep.

If you have any questions regarding the informed consent form or other items of this patient information form, please feel free to ask the research team or the independent expert (see contact details at the last page).

Thank you for your attention,

Yours sincerely,

The research team

1. **Attachment**

A. Contact details research team and Sierra Leone Ethics and Scientific Review Committee**Annex A: Contact details Research Team**

**Coordinators:**

Masanga Hospital:

Issa Sessay, Research Nurse, Masanga Medical Research Unit

Hanna MJL Hazenberg, MD, Masanga Medical Research Unit

Phone numbers: 0023276481882 or 0023278809787

Lion Heart Medical Centre:

Hassan Bangura, CHO, Lion Heart Medical Centre

Louise K Hoevenaars, MD, Lion Heart Medical Centre

Phone numbers: 0023279230549 or 0023274336507

**Supervising coordinators:**

Jan Henk Dubbink, MD PhD, Medical superintendent Masanga Hospital
Heleen Koudijs, MD, Medical superintendent Yele Hospital

**Independent expert:**

James Bangura, MD Masanga Hospital, phone number 0023276977535

**Who to contact if any complaints:**

Hanna MJL Hazenberg, MD, Masanga Medical Research Unit, 0023278809787

**Contact details Sierra Leone Ethics and Scientific Research Committee**

**GOVERNMENT OF SIERRA LEONE**

Office of the Sierra Leone Ethics and Scientific Review Committee

Ministry of Health and Sanitation

Directorate of Policy, Planning & Information (DPPI)

Youyi Building, Fifth Floor, East Wing

Email address: efoday@mohs.gov.sl

Phone number: +23278366493
