# Supplementary material for: Complicated Odontogenic Infections at 2 District Hospitals in Tonkolili District, Sierra Leone: Protocol for a Prospective Observational Cohort Study (DELAY)
Source: JMIR Res Protoc. 2021 Dec 13;10(12):e33677. doi: 10.2196/33677 (PMC8713131; doi:10.2196/33677)
Supplement: Multimedia Appendix 3 [file resprot_v10i12e33677_app3.docx]

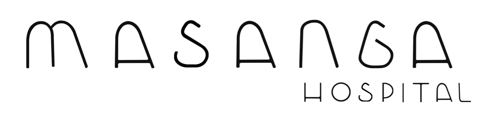


**Informed Consent**

**Investigator Script**

**Informed Consent form for DEntaL Abscess studY (DELAY): Prospective observational cohort study of complicated odontogenic infections in two district hospitals in Tonkolili District, Sierra Leone**

This document is for men and women participating in the **Prospective Observational Cohort Study of Complicated Odontogenic Infections in Two District Hospitals in Tonkolili District, Sierra Leone,** or for legal representatives in case of inability to make an informed decision.

I am (**investigator name**), working for Masanga Hospital/Lion Heart Medical Centre. We are doing research on complications of tooth infections, which is a disease with very concerning consequences. I am going to give you information and invite you to be part of this research. I want you to be aware that you might withdraw your consent/participation without the need to mention a reason, and that it will not result in any disadvantage regarding the medical care you receive.

You decide if you want to be in the study or not. There may be some words that you do not understand. Please ask me to stop as we go through the information and I will take time to explain. If you have questions later, you can ask them to me.

With this research, we want to know what factors play a role when the tooth infection becomes a serious disease. To identify the factors, we will ask you some questions about your health and do some physical examinations, like measuring the size of the swelling present. This won’t hurt you. If laboratory tests results prove positive for HIV and Hepatitis B, you will be informed and assisted to gain access to treatment programs as per standard hospital care. It is important to notice that your care and treatment will not be affected by agreeing to be part in the study or not. We are inviting everyone with a toothache that needs admission to the hospital because of a big infection to be part of this study.

Your participation in this research is entirely voluntary. It is your choice whether to participate or not. Whether you choose to participate or not, all the services you receive at this centre will continue and nothing will change. If you choose not to participate in this research project, you will be offered the same treatment that is routinely offered in this facility for tooth infections that have become severe.

You may change your mind later and stop participating even if you agreed earlier.

We will not be sharing the identity or any personal information of participants in the research.

We are facilitating this research to get more knowledge about the disease which might help us in the future to fight this disease.

Do you have any questions?

Any further inquiry can be addressed to [masangahospitalsl@gmail.com](mailto:masangahospitalsl@gmail.com) or via (+232) 078809787


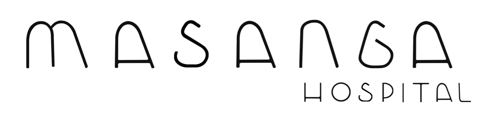


**Informed Consent**

**Patient Form**

**Informed Consent form for DEntaL Abscess studY (DELAY): Prospective Observational Cohort Study of Complicated Odontogenic Infections in Two District Hospitals in Tonkolili District, Sierra Leone**

**I have read the foregoing information, or it has been read to me. I have had the opportunity to ask questions about it and any questions that I have asked have been answered to my satisfaction. I consent voluntarily to participate as a participant in this research.**

**Name of Participant __________________**

Name of person consenting (if not participant) **__________________**

Relationship to participant  **__________________**

**Signature of Participant/caretaker to participate in the study ________________**

**Signature of Participant/caretaker for agreement medical photos _____________­­­­­**

**Date ___________________________ Day/month/year**

**Thumb print of participant/caretaker Thumb print of participant/caretaker**

**For consent participating in study For consent taking medical photo’s**

**(if illiterate) (if illiterate)**

**Statement by the researcher**

I have accurately read out the information sheet to the potential participant, and to the best of my ability made sure that the participant understands.

I confirm that the participant was given an opportunity to ask questions about the study, and all the questions asked by the participant have been answered correctly and to the best of my ability. I confirm that the individual has not been coerced into giving consent, and the consent has been given freely and voluntarily.

**Name of Researcher** **___________________________**

**Signature of Researcher ____________________________**

**Date ____________________________ Day/month/year**


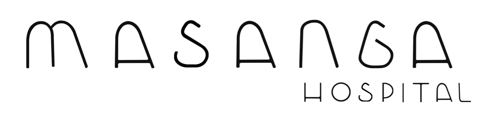


**Informed Consent**

**Researcher Form**

**Informed Consent form for DEntaL Abscess studY (DELAY): Prospective Observational Cohort Study of Complicated Odontogenic Infections in Two District Hospitals in Tonkolili District, Sierra Leone**

**I have read the foregoing information, or it has been read to me. I have had the opportunity to ask questions about it and any questions that I have asked have been answered to my satisfaction. I consent voluntarily to participate as a participant in this research.**

**Name of Participant __________________**

Name of person consenting (if not participant) **__________________**

Relationship to participant  **__________________**

**Signature of Participant/caretaker to participate in the study ________________**

**Signature of Participant/caretaker for agreement medical photos _____________­­­­­**

**Date ___________________________ Day/month/year**

**Thumb print of participant/caretaker Thumb print of participant/caretaker**

**For consent participating in study For consent taking medical photo’s**

**(if illiterate) (if illiterate)**

**Statement by the researcher**

I have accurately read out the information sheet to the potential participant, and to the best of my ability made sure that the participant understands.

I confirm that the participant was given an opportunity to ask questions about the study, and all the questions asked by the participant have been answered correctly and to the best of my ability. I confirm that the individual has not been coerced into giving consent, and the consent has been given freely and voluntarily.

**Name of Researcher ___________________________**

**Signature of Researcher ____________________________**

**Date ____________________________ Day/month/year**
