# Supplementary material for: Complicated Odontogenic Infections at 2 District Hospitals in Tonkolili District, Sierra Leone: Protocol for a Prospective Observational Cohort Study (DELAY)
Source: JMIR Res Protoc. 2021 Dec 13;10(12):e33677. doi: 10.2196/33677 (PMC8713131; doi:10.2196/33677)
Supplement: Multimedia Appendix 4 [file resprot_v10i12e33677_app4.pdf]

Masanga Medical Research Unit
Masanga Hospital, PO box – 44 Magburaka
Tonkolili district, Sierra Leone
E-mail: m.j.gortzak@amc.uva.nl and m.p.grobusch@amc.uva.nl (Cc)

Decision Letter

MMRU Scientific Review Committee

**General information**	
Principal Investigator (PI)	Professor Martin P Grobusch
Corresponding Investigator	Hanna Hazenberg
Date of submission	07-06-2021
Proposal title	DEntaL Abscess studY (DELAY): Prospective observational cohort study of complicated odontogenic infections in two district hospitals in Tonkolili District, Sierra Leone
Acronym	DELAY
SRC number	MMRU-SRC-002-2021

**Overall assessment**	**Score** (OK, n/a, clarification required)
Scientific quality	OK
Scientific interest	OK
Ethical considerations	OK
Project feasibility	OK
Relationship to MMRU research policy	OK
Relationship to national research policy	OK
Other issues	n/a

Decision ☐ Accepted without remarks

X Accepted with remarks*

□ Resubmission following revision

□ Rejected

***Remarks:**

Nice proposal, very necessary to pay more attention to odontological infections in SL. Ambitious project with many objectives. Two brief questions/remarks:

-How will be the WBC performed? Manually or with an automatic cell-counter? If possible, I would advice to also perform a differential WBC (especially if this is not manually performed).

-How will be the pus swab taken? And how will it be stored till and during the shipment?

A proposal that I'm very happy to read. I have treated many severe cases of Ludwig angina and necrotizing fasciitis in my years in Masanga, and for me they have always been amongst the worst and challenging patients I have treated. So, definitely happy to see this moving forward.

Some comments;

- are there local treatment guidelines?

- date of surgery collect also which surgery was done, just incision and drainage, tooth extraction, (repetitive) debridement and/or possible split skin grafting

- after discharge collect 'skingrafted/not grafted', 'duration of hospitalization'.

- at follow up: measurement of mouth opening, since tooth extraction may only be possible in a postponed setting and not at initial treatment of the odontological abscess/infection they present at first with. However it remains the root of the problem but may initially be inaccesible and poses a possible cause of future deterioration.

Amsterdam, 16-06-2021

Place/Date ____

Signature ____

MP Grobusch, SRC Chair and Director, MMRU
